# Performance of rapid diagnostic test, blood-film microscopy and PCR for the diagnosis of malaria infection among febrile children from Korogwe District, Tanzania

**DOI:** 10.1186/s12936-016-1450-z

**Published:** 2016-07-26

**Authors:** Coline Mahende, Billy Ngasala, John Lusingu, Tai-Soon Yong, Paminus Lushino, Martha Lemnge, Bruno Mmbando, Zul Premji

**Affiliations:** 1 Korogwe Research Station, Tanga Centre, National Institute for Medical Research, P. O. Box 5004, Tanga, Tanzania; 2 Department of Medical Entomology and Parasitology, School of Public Health, Muhimbili University of Health and Allied Sciences, P. O. Box 65001, Dar es Salaam, Tanzania; 3 Department of International Health, Microbiology and Immunology, University of Copenhagen, Copenhagen, Denmark; 4 Department of Environmental Medical Biology, Institute of Tropical Medicine, Yonsei University College of Medicine, Seoul, 03722 Republic of Korea

**Keywords:** Malaria, *Plasmodium falciparum*, Malaria rapid diagnostic test, Microscopy, Polymerase chain reaction (PCR), Outpatient, Children, Febrile, Korogwe, Tanzania

## Abstract

**Background:**

Rapid diagnostic tests (RDT) and light microscopy are still recommended for diagnosis to guide the clinical management of malaria despite difficult challenges in rural settings. The performance of these tests may be affected by several factors, including malaria prevalence and intensity of transmission. The study evaluated the diagnostic performance of malaria RDT, light microscopy and polymerase chain reaction (PCR) in detecting malaria infections among febrile children at outpatient clinic in Korogwe District, northeastern Tanzania.

**Methods:**

The study enrolled children aged 2–59 months with fever and/or history of fever in the previous 48 h attending outpatient clinics. Blood samples were collected for identification of *Plasmodium falciparum* infection using histidine-rich-protein-2 (HRP-2)-based malaria RDT, light microscopy and conventional PCR.

**Results:**

A total of 867 febrile patients were enrolled into the study. Malaria-positive samples were 85/867 (9.8 %, 95 % CI, 7.9–12.0 %) by RDT, 72/867 (8.3 %, 95 % CI, 6.5–10.1 %) by microscopy and 79/677 (11.7 %, 95 % CI, 9.3–14.3 %) by PCR. The performance of malaria RDT compared with microscopy results had sensitivity and positive predictive value (PPV) of 88.9 % (95 % CI, 79.3–95.1 %) and 75.3 % (95 % CI, 64.8–84.0 %), respectively. Confirmation of *P. falciparum* infection with PCR analysis provided lower sensitivity and PPV of 88.6 % (95 % CI, 79.5–94.7 %) and 84.3 % (95 % CI, 74.7–91.4 %) for RDT compared to microscopy.

**Conclusion:**

Diagnosis of malaria infection is still a challenge due to variation in results among diagnostic methods. HRP-2 malaria RDT and microscopy were less sensitive than PCR. Diagnostic tools with high sensitivity are required in areas of low malaria transmission.

## Background

Despite a remarkable reduction in *Plasmodium falciparum* transmission across sub-Saharan Africa, malaria still remains a public health problem, especially among children aged under 5 years [1, 2]. In line with the decline, there is an urgent need for improved diagnostic tools in order to prepare for resurgence [3]. It is crucial to understand the performance of different malaria diagnostic tests in different settings during this new epidemiological context.

Fever has been the major complaint amongst children presenting at outpatient clinics, with malaria being the possible aetiology for such febrile illnesses [4]. Integrated management of childhood illness (IMCI) guidelines was developed by the World Health Organization (WHO) to improve clinical management of febrile illnesses in developing countries [5]. However, in 2010 as a result of over-diagnosis coupled with over-prescription of anti-malarials, WHO updated the guidelines specifically to improve diagnosis of malaria infection by restricting anti-malarial treatment to patients with positive test results [6, 7]. Despite this recommendation the performance of malaria diagnostic tools is affected by several factors, including low transmission, low parasite density and lack of qualified technicians for microscopy [8, 9]. This underscores the need to investigate further accurate diagnosis of malaria infection in febrile patients towards optimal use of anti-malarials, case management of asymptomatic cases and disease surveillance.

Despite its low sensitivity and limited availability, microscopy still remains the gold standard for malaria diagnosis. Malaria microscopy is time consuming, requires skilled laboratory technicians and is often subject to unreliable results from different laboratories [10]. With the availability of malaria rapid diagnostic testing (RDT), WHO recommended its use for prompt and accurate confirmation of *P. falciparum* infection in settings with limited laboratory facilities [11]. Both microscopy and malaria RDT have limited detection threshold, especially in situations with low parasitaemia [12, 13]. Polymerase chain reaction (PCR) is regarded as one of few most sensitive molecular techniques for detecting parasites at limits of 0.01–0.2 parasites/μL of blood [14, 15]. As with microscopy and malaria RDT, there have been different PCR assays reporting varying sensitivities and specificities [16, 17]. Nonetheless, despite being highly sensitive, PCR is expensive and cannot be utilized in routine practice in resource-limited settings.

The performance of malaria diagnostic tests tends to vary depending on different settings of malaria transmission. The study evaluated the diagnostic performance of malaria RDT, light microscopy and conventional PCR in detecting *P. falciparum* malaria infection among febrile children at outpatient clinics in Korogwe District, northeastern Tanzania.

## Methods

### Study site and population

Korogwe District is located in an area with differing low to moderate *P. falciparum* malaria transmission in Tanga region, northeastern Tanzania [18]. The environment is characterized by daily temperatures varying from 18 to 20 °C during the rainy season and 26–30 °C during the dry season. The annual rainfall ranges from 700 to 1000 mm with long rainy seasons extending from March to May. The majority of the inhabitants reside in rural settings, practicing subsistence farming and informal trade. Korogwe District Hospital (KDH) is a secondary health care facility receiving approximately 6000 outpatient visits from a population of around 73,275 children under the age of 5 years living in Korogwe District and nearby villages from Handeni District [19]. The prevalence of fever cases with *P. falciparum* parasitaemia among children under 5 years of age from the community in both lowland and highland villages between 2009 and 2010 was below 10 % [20].

### Enrolment of participants

Sick children presenting at KDH outpatient clinic between monday and friday of every week were assessed for study eligibility. The inclusion criteria were: children aged between 2 and 59 months presenting at KDH with a history of fever in the previous 48 h or with measured axillary body temperature >37.5 °C at presentation. The visit should be their first consultation for a present problem. Exclusion criteria included severe and chronic illnesses, intake of antimicrobial and/or anti-malarial drugs within the previous 7 days, planned admissions (e.g., elective surgery), and trauma/injury.

### Study procedure and laboratory analyses

#### Clinical examination and demographic information

Medical history and clinical examination were performed on each patient by the study clinician and information was entered into a standardized case report form. This included demographic information, clinical history, vital signs, body weight, signs of dehydration, and neurological and physical examinations (skin, abdominal, ear, mouth, throat). Clinical diagnosis of malaria was done according to IMCI guidelines [5]. A maximum of 1 mL venous blood was drawn from every patient for laboratory investigations.

#### Malaria detection using RDT

Histidine rich protein 2 (HRP-2)-based RDT, paraHIT^®^f (Span Diagnostics Limited, India) was used for detection of malaria infection. The test detects HRP-2 antigen from *P. falciparum* only. The tests were performed and interpreted following manufacturer’s instructions. The malaria RDT test results were provided directly to patients and assisted the clinician on clinical decision.

#### Malaria detection using blood slide microscopy

Thick and thin blood smears were prepared (in duplicate) from the blood collected in the ethylene diamine tetra acetic acid (EDTA) tubes. The thin film was fixed with methanol and blood slides were stained with a 5 % Giemsa solution for identification and quantitation of asexual *P. falciparum* and other *Plasmodium* species. The blood slides were read by two expert microscopists and in case of discrepancy, a third reading was performed. Asexual parasites were counted against 200 (500 if parasite count was <10) white blood cells. A blood slide was considered negative for *Plasmodium* species if no parasites were seen in at least 100 oil-immersion, high-power fields on the thick film.

#### Malaria detection using PCR

Three drops, each containing 50 μL of EDTA blood were spotted on a pre-made filter paper (Whatman 3MM, Maidstone, UK) and allowed to dry at room temperature. The filter papers were placed in zip-locked, plastic bags containing silica gel to preserve DNA integrity and stored at −20 °C and then transported to Republic of Korea for molecular analysis. DNA was extracted from half-segment of the filter spot using QIAamp DNA Blood Mini Kit (Qiagen, Hilden, Germany) as described by the manufacturer, and eluted in a final volume of 100 μL. The extracted DNA was analysed using *P. falciparum* species-specific PCR targeting mitochondrial genome 18S as previously described by Kho et al. [21]. The parasite DNA amplification was performed using conventional PCR 96 well Thermal Cycler (Applied Biosystems, Carlsbad, CA, USA). The PCR products were analysed in 1.5 % ethidium bromide stained UltraPure™ agarose gel (Invitrogen) with a Gene ruler™ 100 bp DNA ladder (TaKara Bio Inc, Shiga, Japan). The gels were visualized under UV transilluminator from BIO-RAD.

### Quality control and assessment

Laboratory investigations were carried out at Korogwe Research Laboratory of the National Institute for Medical Research (Tanzania). PCR analysis was performed at the Institute of Tropical Medicine, Yonsei University (Republic of Korea). Quality control was conducted for all laboratory methods and procedures according to standard guidelines and manufacturers’ instructions. During the study, laboratory microscopists participated in the parasitology external quality assessment programme with the National Institute for Communicable Diseases (NICD).

### Data management and statistical analyses

Data were double entered and validated using Microsoft Access database. Data analysis was performed using STATA (Stata Corp LP, College Station, Texas, USA). Variables were summarized as frequencies and percentages, medians and inter-quartile ranges as appropriate. Odds ratios and 95 % confidence intervals (CI) were calculated as appropriate and the value of *p* < 0.05 was considered statistically significant. The performance of each diagnostic test method was calculated by means of sensitivity, specificity, positive predictive value (PPV), and negative predictive value (NPV) using conventional PCR as a reference standard.

## Results

**Fig. 1:**
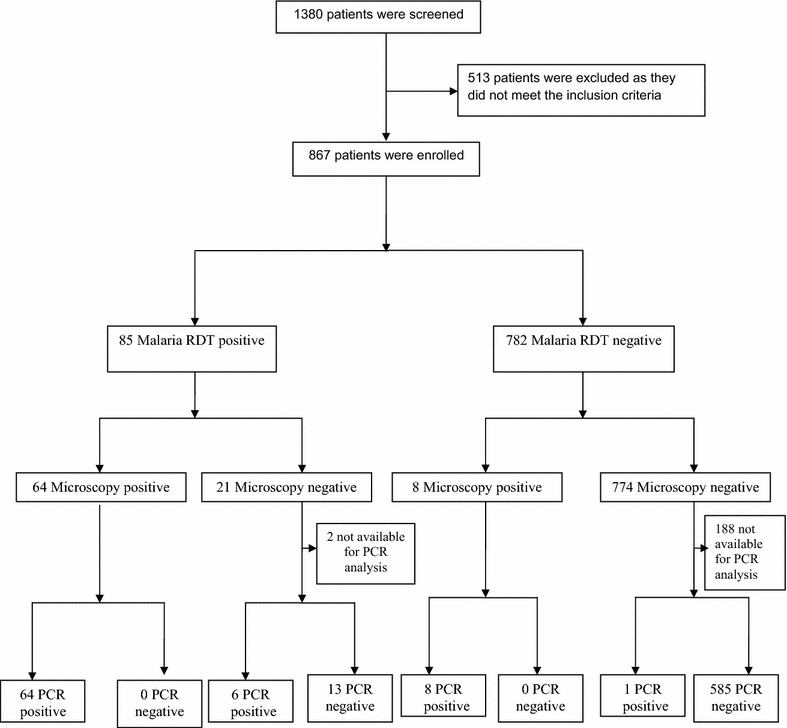
Study flow diagram showing patient enrolment and diagnostics performed

A total of 1380 sick children attending the outpatient clinic at KDH from January to October 2013 were screened; 867/1380 (62.8 %) met the inclusion criteria and were enrolled. The median age was 15.1 months (interquartile range (IQR): 8.6–29.9) and girls accounted for 408/867 (47.1 %) of patients (Table 1). Samples from all patients were tested for malaria infection using HRP-2-based RDT and 85/867 (9.8 %, 95 % CI, 7.9–12.0 %) were positive. Seventy-two samples (8.3 %, 95 % CI, 6.5–10.1 %) were positive for *P. falciparum* by microscopy with geometric mean parasitaemia of 37,635 (4715–12,959) parasites/μL. Dried blood spots samples on filter paper for PCR analysis were prepared and performed from 677/867 (78.1 %) patients, of which 79/677 (11.7 %, 95 % CI, 9.3–14.3 %) were positive for *P. falciparum*. Samples from 190/867 (21.9 %) patients were not available for PCR analysis; 153/190 (80.5 %) patients did not have sufficient blood for spotting on a pre-made filter paper and 37/190 (19.5 %) patients had low yield DNA extracted from dried blood spots on filter paper. The complete flow of the performed diagnostic tests and results are outlined in Fig. 1.

**Table 1:** Patient characteristics

Patient characteristics	Total (%)
Gender	
Girls	408 (47.1)
Boys	459 (52.9)
Age	
2–11 months	356 (41.1)
12–35 months	370 (42.7)
36–59 months	141 (16.3)
Duration of fever in days, median (IQR)	2 (1–2)
Axillary temperature ≥37.5 °C (IQR)	38.1 (37.5–38.7)
Parasite density levels per µL blood (n = 72)	
<1000	3 (4.2)
1000–100,000	43 (59.7)
100,000–200,000	9 (12.5)
>200,000	17 (23.6)

*IQR* interquartile range

**Fig. 2:**
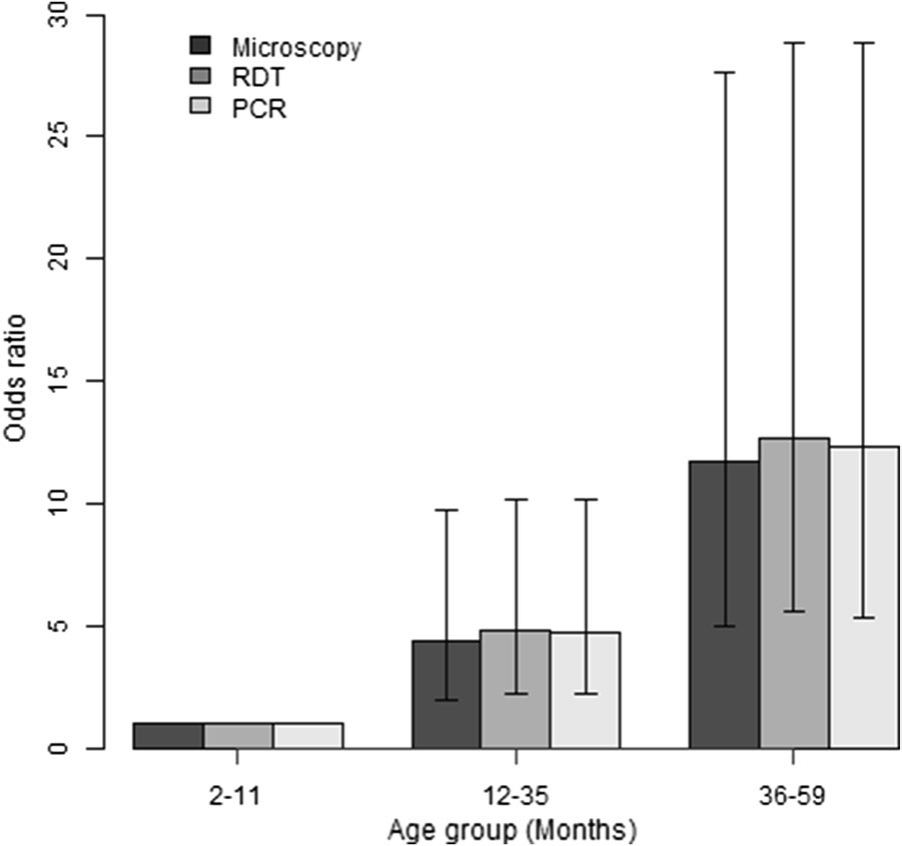
Distribution of *Plasmodium falciparum* prevalence by age group and by diagnostic tools. Compared to children <12 months, the prevalence increased significantly (p < 0.001) by age group in all three diagnostic methods

The trends of malaria positivity by age group were similar between the three diagnostic methods, and the odds ratio of being diagnosed positive increased significantly by age (p < 0.001 for all three diagnostic tools), Fig. 2. The odds ratio in age group 2–12 months was 4.4 (95 % CI, 1.99–9.74 %) while that of children aged 36–59 months was 11.76 (95 % CI, 5.0–27.65 %) compared to that of children under 12 months old.

### Performance of malaria RDT compared with microscopy results

Among samples with positive malarial parasite antigen by RDT, 21/85 (24.7 %) were confirmed not having *P. falciparum* parasitaemia by light microscopy (Table 2). Of the 782/867 (90.2 %) samples that were malaria RDT negative, eight had *P. falciparum* by microscopy with parasitaemia ranging between 1280 and 632,400 parasites/μL. The sensitivity and specificity of malaria RDT was 88.9 % (95 % CI, 79.3–95.1 %) and 97.4 % (95 % CI, 96.0–98.4 %), respectively, with corresponding positive and negative predictive values of 75.3 % (95 % CI, 64.8–84.0 %) and 99.0 % (95 % CI, 98.0–99.6 %) (Table 2).

**Table 2:** Sensitivity, specificity, positive, and negative predictive value of HRP2-based malaria RDT against microscopy results

RDT	Microscopy		Sensitivity	Specificity	PPV	NPV
	Positive	Negative	% [95 % CI]	% [95 % CI]	% [95 % CI]	% [95 % CI]
Positive	64	21	88.9 (79.3–95.1)	97.4 (96.0–98.4)	75.3 (64.8–84.0)	99.0 (98.0–99.6)
Negative	8	774				

### Confirmation of *Plasmodium falciparum* infection with PCR analysis

Of the 21 malaria RDT-positive samples but microscopy negative, six samples were detected positive by PCR while two samples were not available for analysis. Eight samples that were RDT negative but microscopy positive were confirmed having *P. falciparum* species by PCR. One sample that was both RDT and microscopy negative was detected positive by PCR. The sensitivity (88.6 %) and PPV (84.3 %) were lower for RDT compared to microscopy (Table 3).

**Table 3:** Sensitivity, specificity, positive, and negative predictive value of HRP2-based malaria RDT and microscopy against PCR results

	PCR		Sensitivity	Specificity	PPV	NPV
	Positive	Negative	% [95 % CI]	% [95 % CI]	% [95 % CI]	% [95 % CI]
RDT						
Positive	70	13	88.6 (79.5–94.7)	97.8 (96.3–98.8)	84.3 (74.7–91.4)	98.5 (97.1–99.3)
Negative	9	585				
Microscopy						
Positive	72	0	91.1 (82.6–96.4)	100.0 (99.4–100.0)	100.0 (95.0–100.0)	98.8 (97.6–99.5)
Negative	7	598				

## Discussion

This study evaluated the performance of different diagnostic tools in detecting malaria infection among febrile children attending outpatient clinic at Korogwe District. PCR performed better than blood slide expert microscopy and HRP-2-based malaria RDT. Overall, malaria prevalence was low and significantly associated with age where the majority of patients were over 12 months old. Infants appear to be relatively protected from malaria (and other infections) for the first 6 months of life, mainly due to acquiring maternal antibodies and the presence of foetal haemoglobin [22–24]. Three-quarters of malaria patients had low levels of parasitaemia (<200,000 parasites/μL).

The use of HRP-2-based malaria RDT in the current study resulted in over-diagnosis of malaria infection in 15 % of patients. This might suggest the presence of recent malaria infection. When compared with expert microscopy and PCR, the sensitivity value of malaria RDT was below the ≥95 % threshold recommended by WHO. These findings are similar to those reported by Shakely and colleagues on a hospital-based study conducted in Zanzibar, an area of low malaria transmission [25]. HRP-2-based malaria RDT is known for detecting malaria antigens that continue to circulate in blood almost 2 months after treatment of a malaria episode [26]. This is the most probable explanation for the false-positive results and sub-microscopic infections that were observed in the study. In the current study, eight patients had false-negative malaria RDT results despite having moderate to high levels of parasitaemia. This could be due to malaria parasites expressing low level of target antigen, deleted *pfhrp2* gene, prozone effect or other factors, which could not be established [27–32]. However, current information from WHO indicates that in most settings, genetic mutations (deletion of *pfhrp2*/*pfhrp3)* in parasites are not likely to be the main cause of false-negative results [33]. Therefore, further research is required to determine the true prevalence of these mutations. Despite these limitations, HRP-2-based malaria RDTs remain the preferred choice, mainly in settings with limited microscopy facilities, due to easy availability and low cost [34]. There are continued efforts to improve sensitivity and specificity of malaria RDTs for rapid and better management of malaria cases. Clinicians are advised to investigate other causes of febrile episodes despite a positive malaria RDT test. Different types of infections are common in children from resource-limited areas that lack adequate sanitation and clean water supply. Therefore, it is very likely that children attending outpatient clinics from rural settings present with multiple infections [35].

Identification of sub-microscopic infection by PCR has demonstrated that expert microscopy can miss detection of malaria in patients having low parasite densities hence leading to false-negative results. As malaria transmission declines, cases of sub-microscopic infection in both symptomatic and asymptomatic persons is likely to increase [36]. PCR and other molecular techniques are indicated to be the most sensitive diagnostic tool than microscopy and RDT [37, 38]. However, its utility in routine practice remains a concern especially at this era towards malaria elimination.

The study had a number of limitations. Firstly, some of the patients may have been on anti-malarial therapy despite verbal confirmation provided by their parents/guardians on the prior use of anti-malarial drugs. This would have contributed to the false-positive RDT results. Secondly, the malaria RDT and PCR diagnostic tools used in the current study could not detect other species of *Plasmodium* apart from *P. falciparum*. This could have missed a few malaria infections of other species, such as *P. malariae* and *P. ovale* that have been reported in the country [29, 39, 40]. Both *P. malariae* and *P. ovale* occur more commonly as mixed infections with *P. falciparum* [40–42]. Lastly, the study may have biased the estimated prevalence of sub-microscopic infections due to the unavailability of nearly a quarter of patient samples for PCR analysis.

## Conclusion

The prevalence of malaria infection was low among outpatient children from Korogwe District. Diagnosis of malaria infection is still a challenge due to variation in results among diagnostic methods. HRP-2 malaria RDT and microscopy were less sensitive than PCR. Diagnostic tools with high sensitivity are required in areas of low malaria transmission.

## Abbreviations

RDT: rapid diagnostic test
PCR: polymerase chain reaction
HRP-2: histidine-rich-protein-2
CI: confidence interval
PPV: positive predictive value
IMCI: Integrated Management of Childhood Illness
WHO: World Health Organization
KDH: Korogwe District Hospital
EDTA: ethylene diamine tetra acetic acid
NICD: National Institute for Communicable Diseases
NPV: negative predictive value
IQR: interquartile range

## References

[1] WHO: World malaria report. Geneva: World Health Organization; 2015.

[2] Murray CJ, Rosenfeld LC, Lim SS, Andrews KG, Foreman KJ, Haring D (2012). Global malaria mortality between 1980 and 2010: a systematic analysis. Lancet.

[3] Strom GE, Haanshuus CG, Fataki M, Langeland N, Blomberg B (2013). Challenges in diagnosing paediatric malaria in Dar es Salaam, Tanzania. Malar J..

[4] Lundgren IS, Heltshe SL, Smith AL, Chibwana J, Fried MW, Duffy PE (2015). Bacteremia and malaria in Tanzanian children hospitalized for acute febrile illness. J Trop Pediatr.

[5] WHO. Intergrated Management of Childhood Illness. Department of Child and Adolescent Health and Development (CAH). Geneva: World Health Organization; 2005.

[6] Reyburn H, Mbatia R, Drakeley C, Carneiro I, Mwakasungula E, Mwerinde O (2004). Overdiagnosis of malaria in patients with severe febrile illness in Tanzania: a prospective study. BMJ.

[7] WHO. Guidelines for the treatment of malaria. 2nd ed. Geneva, Switzerland: World Health Organization; 2010.

[8] Okell LC, Bousema T, Griffin JT, Ouedraogo AL, Ghani AC, Drakeley CJ (2012). Factors determining the occurrence of submicroscopic malaria infections and their relevance for control. Nat Commun..

[9] Ngasala B, Mubi M, Warsame M, Petzold MG, Massele AY, Gustafsson LL (2008). Impact of training in clinical and microscopy diagnosis of childhood malaria on antimalarial drug prescription and health outcome at primary health care level in Tanzania: a randomized controlled trial. Malar J..

[10] Wilson ML (2013). Laboratory diagnosis of malaria: conventional and rapid diagnostic methods. Arch Pathol Lab Med.

[11] WHO. Universal access to malaria diagnostic testing. Geneva: World Health Organization; 2012.

[12] Mtove G, Nadjm B, Amos B, Hendriksen IC, Muro F, Reyburn H (2011). Use of an HRP2-based rapid diagnostic test to guide treatment of children admitted to hospital in a malaria-endemic area of north-east Tanzania. Trop Med Int Health..

[13] Bejon P, Andrews L, Hunt-Cooke A, Sanderson F, Gilbert SC, Hill AV (2006). Thick blood film examination for *Plasmodium falciparum* malaria has reduced sensitivity and underestimates parasite density. Malar J..

[14] Cordray MS, Richards-Kortum RR (2012). Emerging nucleic acid-based tests for point-of-care detection of malaria. Am J Trop Med Hyg.

[15] Mosha JF, Sturrock HJ, Greenhouse B, Greenwood B, Sutherland CJ, Gadalla N (2013). Epidemiology of subpatent *Plasmodium falciparum* infection: implications for detection of hotspots with imperfect diagnostics. Malar J..

[16] Xu W, Morris U, Aydin-Schmidt B, Msellem MI, Shakely D, Petzold M (2015). SYBR Green real-time PCR-RFLP assay targeting the Plasmodium cytochrome B gene—a highly sensitive molecular tool for malaria parasite detection and species determination. PLoS ONE.

[17] Lo E, Zhou G, Oo W, Afrane Y, Githeko A, Yan G (2015). Low parasitemia in submicroscopic infections significantly impacts malaria diagnostic sensitivity in the highlands of Western Kenya. PLoS ONE.

[18] Mmbando BP, Vestergaard LS, Kitua AY, Lemnge MM, Theander TG, Lusingu JP (2010). A progressive declining in the burden of malaria in north-eastern Tanzania. Malar J..

[19] National Bureau of Statistics Ministry of Finance Tanzania. Tanzania: Population and housing census; 2013.

[20] Rutta AS, Francis F, Mmbando BP, Ishengoma DS, Sembuche SH, Malecela EK (2012). Using community-owned resource persons to provide early diagnosis and treatment and estimate malaria burden at community level in north-eastern Tanzania. Malar J..

[21] Kho WG, Chung JY, Sim EJ, Kim MY, Kim DW, Jongwutiwes S (2003). A multiplex polymerase chain reaction for a differential diagnosis of *Plasmodium falciparum* and *Plasmodium vivax*. Parasitol Int.

[22] Riley EM, Wagner GE, Akanmori BD, Koram KA (2001). Do maternally acquired antibodies protect infants from malaria infection?. Parasite Immunol.

[23] Pasvol G, Weatherall DJ, Wilson RJ (1977). Effects of foetal haemoglobin on susceptibility of red cells to *Plasmodium falciparum*. Nature.

[24] McGregor IA (1984). Epidemiology, malaria and pregnancy. Am J Trop Med Hyg.

[25] Shakely D, Elfving K, Aydin-Schmidt B, Msellem MI, Morris U, Omar R (2013). The usefulness of rapid diagnostic tests in the new context of low malaria transmission in Zanzibar. PLoS ONE.

[26] Bisoffi Z, Sirima SB, Menten J, Pattaro C, Angheben A, Gobbi F (2010). Accuracy of a rapid diagnostic test on the diagnosis of malaria infection and of malaria-attributable fever during low and high transmission season in Burkina Faso. Malar J..

[27] Koita OA, Doumbo OK, Ouattara A, Tall LK, Konare A, Diakite M (2012). False-negative rapid diagnostic tests for malaria and deletion of the histidine-rich repeat region of the hrp2 gene. Am J Trop Med Hyg.

[28] Cheng Q, Gatton ML, Barnwell J, Chiodini P, McCarthy J, Bell D (2014). *Plasmodium falciparum* parasites lacking histidine-rich protein 2 and 3: a review and recommendations for accurate reporting. Malar J..

[29] Ishengoma DS, Shayo A, Mandara CI, Baraka V, Madebe RA, Ngatunga D (2016). The role of malaria rapid diagnostic tests in screening of patients to be enrolled in clinical trials in low malaria transmission settings. Health Syst Policy Res..

[30] Kumar N, Pande V, Bhatt RM, Shah NK, Mishra N, Srivastava B (2013). Genetic deletion of HRP2 and HRP3 in Indian *Plasmodium falciparum* population and false negative malaria rapid diagnostic test. Acta Trop.

[31] Gamboa D, Ho MF, Bendezu J, Torres K, Chiodini PL, Barnwell JW (2010). A large proportion of *P. falciparum* isolates in the Amazon region of Peru lack pfhrp2 and pfhrp3: implications for malaria rapid diagnostic tests. PLoS ONE.

[32] Johansson EW, Kitutu FE, Mayora C, Awor P, Peterson SS, Wamani H (2016). It could be viral but you don’t know, you have not diagnosed it: health worker challenges in managing non-malaria paediatric fevers in the low transmission area of Mbarara District, Uganda. Malar J..

[33] Global Malaria Programme. False-negative RDT results and implications of new reports of *P. falciparum* histidine-rich protein 2/3 gene deletions. Geneva: World Health Organization; 2016.

[34] Harchut K, Standley C, Dobson A, Klaassen B, Rambaud-Althaus C, Althaus F (2013). Over-diagnosis of malaria by microscopy in the Kilombero Valley, Southern Tanzania: an evaluation of the utility and cost-effectiveness of rapid diagnostic tests. Malar J..

[35] Bisoffi Z, Sirima SB, Meheus F, Lodesani C, Gobbi F, Angheben A (2011). Strict adherence to malaria rapid test results might lead to a neglect of other dangerous diseases: a cost benefit analysis from Burkina Faso. Malar J..

[36] McMorrow ML, Aidoo M, Kachur SP (2011). Malaria rapid diagnostic tests in elimination settings—can they find the last parasite?. Clin Microbiol Infect.

[37] Golassa L, Enweji N, Erko B, Aseffa A, Swedberg G (2013). Detection of a substantial number of sub-microscopic *Plasmodium falciparum* infections by polymerase chain reaction: a potential threat to malaria control and diagnosis in Ethiopia. Malar J..

[38] Mwingira F, Genton B, Kabanywanyi AN, Felger I (2014). Comparison of detection methods to estimate asexual *Plasmodium falciparum* parasite prevalence and gametocyte carriage in a community survey in Tanzania. Malar J..

[39] Mmbando BP, Segeja MD, Msangeni HA, Sembuche SH, Ishengoma DS, Seth MD (2009). Epidemiology of malaria in an area prepared for clinical trials in Korogwe, north-eastern Tanzania. Malar J..

[40] Manjurano A, Okell L, Lukindo T, Reyburn H, Olomi R, Roper C (2011). Association of sub-microscopic malaria parasite carriage with transmission intensity in north-eastern Tanzania. Malar J..

[41] Mboera LE, Kamugisha ML, Rumisha SF, Kisinza WN, Senkoro KP, Kitua AY (2008). Malaria and mosquito net utilisation among schoolchildren in villages with or without healthcare facilities at different altitudes in Iringa District, Tanzania. Afr Health Sci..

[42] Doctor SM, Liu Y, Anderson OG, Whitesell AN, Mwandagalirwa MK, Muwonga J (2016). Low prevalence of *Plasmodium malariae* and *Plasmodium ovale* mono-infections among children in the Democratic Republic of the Congo: a population-based, cross-sectional study. Malar J..

